# Spontaneous Remission in an Older Patient with Relapsed* FLT3* ITD Mutant AML

**DOI:** 10.1155/2016/1259759

**Published:** 2016-12-29

**Authors:** Pankit Vachhani, Jason H. Mendler, Andrew Evans, George Deeb, Petr Starostik, Paul K. Wallace, Eunice S. Wang

**Affiliations:** ^1^Leukemia Service, Department of Medicine, Roswell Park Cancer Institute, Buffalo, NY, USA; ^2^Department of Medicine, James P. Wilmot Cancer Institute, University of Rochester Medical Center, Rochester, NY, USA; ^3^Department of Pathology and Laboratory Medicine, University of Rochester Medical Center, Rochester, NY, USA; ^4^Department of Pathology and Laboratory Medicine, Emory University, Atlanta, GA, USA; ^5^Department of Pathology, Immunology, and Laboratory Medicine, University of Florida, Gainesville, FL, USA; ^6^Department of Pathology and Laboratory Medicine, Roswell Park Cancer Institute, Buffalo, NY, USA

## Abstract

Spontaneous remission (SR) of acute myeloid leukemia (AML) is a very rare phenomenon. AML characterized by* FLT3* internal tandem duplication (*FLT3* ITD) is typically associated with an aggressive clinical course with rapid progression, relapse, and short overall survival in the absence of transplantation. We report here the first case of SR of* FLT3* ITD mutant AML in the literature. Our patient was an elderly woman with relapsed* NPM1* and* FLT3* ITD mutant AML whose disease underwent SR for a brief duration without precipitating cause. We review the potential immune mechanisms underlying SR in AML and discuss the implications for novel immunotherapeutic approaches for* FLT3* mutant AML.

## 1. Introduction

Spontaneous remission (SR) of acute myeloid leukemia (AML) is a very rare clinical phenomenon that was first described in 1878 by Eisenlohr [1]. It denotes either partial or complete morphologic disappearance of AML without administration of antileukemic therapy [2, 3]. About 100 cases were reported up to 1955 while less than 10 reports were published between 1955 and 1985 [4–7]. Overall, 46 eligible cases were found in the modern English literature between 1950 and 2014 [4]. Here, we present the first reported case of a 73 year-old woman with relapsed* FLT3* ITD mutant AML whose disease underwent spontaneous complete remission without interim therapy.

AML is a biologically heterogeneous myeloid malignancy with diverse disease subsets defined by distinct cytogenetic and molecular features. Nearly 30% of AML cases are characterized by molecular abnormalities in the FMS-like tyrosine kinase-3 (*FLT3*) gene, known as* FLT*3 mutant AML [8, 9]. The majority of these mutations involve internal tandem duplications (ITD) of the juxtamembrane domain-coding sequence of* FLT3*, leading to constitutive activation of FLT3 receptors and triggering of multiple downstream signaling pathways (such as JAK/STAT, Raf/MEK/ERK, and PI3K/Akt), which promote tumor growth [10–12]. In general, the presence of* FLT3* ITD mutation in AML connotes worse outcomes [13, 14]. Indeed, despite achieving complete remission following induction chemotherapy, the majority of patients with normal cytogenetics and* FLT3* ITD mutant AML experience significantly shortened remission duration, lower salvage rate in first remission, and decreased overall survival [14–17]. While the advent of FLT3 inhibitors may change these outcomes for the better, the only current curative modality for* FLT3* ITD mutant AML patients remains allogeneic stem cell transplantation [11, 13, 14].

In this context, our case presents a fascinating account of a rare phenomenon of SR in an otherwise aggressive molecular subset of AML.

## 2. Case Presentation

The patient was a 73-year-old woman with a prior medical history of hypertension who presented with three weeks of dyspnea, nausea, loose stools, and fatigue. Examination was unremarkable. However, complete blood count (CBC) revealed leukocytes of 240,000/*μ*L, hemoglobin of 6.2 g/dL, and platelets of 119,000/*μ*L (Table 1). Peripheral smear showed many promonocytes and a few immature blasts containing rare Auer rods consistent with a diagnosis of AML. Emergent leukopheresis and treatment with hydroxyurea were initiated. Bone marrow biopsy and aspirate showed 73% myeloblasts and immature promonocytes expressing CD13, CD15, CD33, CD45, CD64, CD123, and HLA-DR by immunohistochemistry (IHC) and flow cytometry (FC). Cytogenetic analysis showed a normal karyotype.* FLT-3* ITD and exon 12 mutation of the nucleophosmin-1* (NPM1)* gene were detected (data not shown). Findings were consistent with* NPM1*-mutated AML of FAB M5 subtype.

Induction chemotherapy with standard-dose cytarabine (100 mg/m^2^ once daily × 7 days) and daunorubicin (45 mg/m^2^ once daily × 3 days) was initiated. The patient achieved a complete remission (CR) based on count recovery and repeat bone marrow aspirate showing no blasts or promonocytes. At the time of morphologic CR, no* FLT3* mutation was identified by quantitative PCR in the marrow; however, exon 12 mutation of* NPM1* was identified in a very small fraction of the cells (signal was below the 10% sensitivity control). Allogeneic stem cell transplantation was offered given the high risk for disease progression, but patient declined. She subsequently received two cycles of high-dose cytarabine (1.5 g/m^2^ once daily × 6 days), which she tolerated well with complete count recovery.

**Figure 1:**
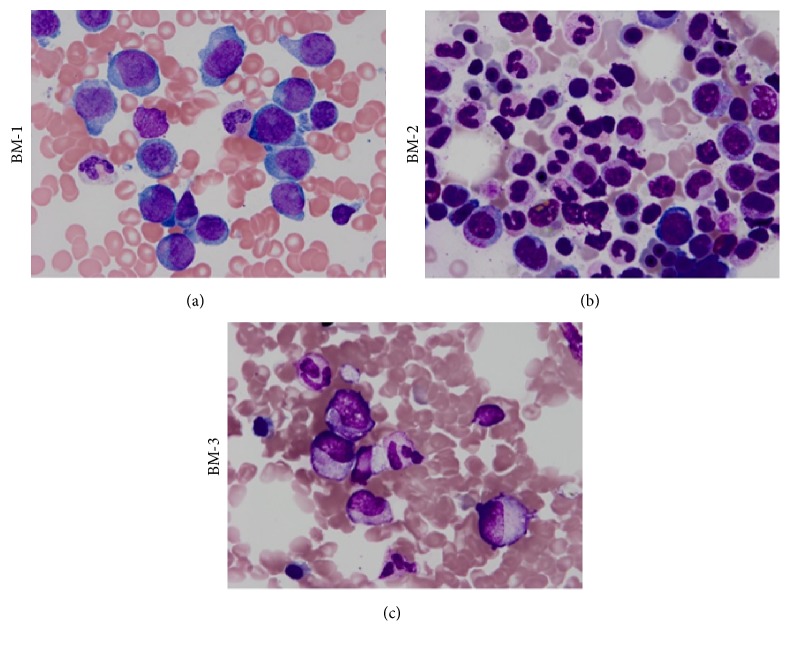
Bone marrow morphology. (a) At time of first detected disease relapse (BM-1); (b) first detection of spontaneous remission (BM-2); (c) second detection of spontaneous remission (BM-3). All marrow aspirate slides were photographed at magnification ×1000.

Six months after completion of consolidation therapy and eight months from her diagnosis, the patient was noted on routine follow-up to have mild leukopenia of 3,300/*μ*L and thrombocytopenia of 115,000/*μ*L. Repeat bone marrow biopsy and aspirate (Figure 1(a)) showed 64% myeloblasts which expressed a slightly different immunophenotype than originally (CD13, CD33, CD15, CD58, CD117, and HLA-DR). Cytogenetics revealed a new abnormality in the form of del (15q) in 19/20 analyzed cells. Both* NPM1* and* FLT3* ITD mutations were identified with a FLT3 ITD allele/wild-type ratio of 0.36.

The patient was referred to a clinical trial of an experimental FLT3 inhibitor. She underwent screening bone marrow biopsy and aspirate (Figure 1(b)) on the contralateral side from BM-1 approximately one week apart. Surprisingly, this demonstrated cellular marrow with 3% blasts without Auer rods and no evidence of AML by marrow IHC and FC. Cytogenetics revealed del (15q) in 1/20 analyzed cells. Molecular analyses detected the presence of a very small amount of* NPM1* mutation in the blood and marrow and a minimal amount of FLT3 with an allelic ratio of 0.005 in the peripheral blood only. The patient reported no interim known or suspected infections or administration of blood products between BM-1 and BM-2. Given these incongruent marrow findings, another bone marrow biopsy and aspirate procedure (Figure 1(c)) was performed on the ipsilateral side as BM-1 another week later. This specimen also showed no evidence of AML by morphology or FC with 2% blasts. Cytogenetics revealed del (15q) in 6/20 analyzed cells. Molecular analyses on marrow aspirate showed detectable* NPM1* and* FLT3* mutations with a FLT3 ITD allele/wild-type ratio of 0.01. To confirm that these marrow samples were indeed from the same patient, genomic DNA was extracted from the BM-1, BM-2, and BM-3 marrow samples followed by amplification of nine short tandem repeat loci (D3S1358, vWA, FGA, D8S1179, D21S11, D18S51, D5S818, D13S317, and D7S820) using the AmpFLSTR® Profiler Plus® PCR Amplification Kit from Life Technologies. All of the samples had identical results at all nine loci, thus confirming that the specimens were from the same individual. Unfortunately the patient was not deemed a candidate for clinical trial of experimental FLT3 inhibitor due to the lack of overt morphological disease, which was an eligibility criterion. She elected to resume observant therapy.

Thirty-five days from first documented SR (BM-2), the patient presented with overt disease recurrence with leukocytes of 29,900/*μ*L and 16% peripheral blasts. Marrow biopsy revealed 84% myeloblasts with del (15q) in all 20 analyzed cells and new del (16q) in 1 of 20 cells. Molecular analyses confirmed the same* NPM1* and* FLT3* ITD mutations with a high allelic ratio of 1.99. The patient decided to forego further treatment and died a few weeks later.

## 3. Discussion

Spontaneous remission of AML is a rare phenomenon documented in the medical literature. To our knowledge, we present here the first reported case of* FLT3* ITD mutant AML undergoing SR. Given the overall prevalence of* FLT3* ITD in AML patients, it is more than likely that some of the prior published cases of SR in AML patients contained other cases of* FLT3* ITD mutant disease which were not discerned due to the lack of available testing. Additionally, the immediate use of chemotherapy after initial diagnosis and at relapse and the innately worse outcomes rendered by mutant* FLT3 *in AML would also have likely contributed to the lack of any prior report of SR. Despite the spontaneous morphologic CR in our patient, it is clear that she had cytogenetic and molecular evidence of AML disease throughout her entire disease course. It is possible that many of the prior reported cases of SR in the literature would not survive the test of today's diagnostic standards. As an example, 21 of the 46 patients reported by Rashidi and Fisher as cases of SR did not even have baseline cytogenetic analysis [4]. While the current definition of complete (and hence spontaneous) remission in AML relies solely on morphologic criteria, there is growing consensus that identification of minimal residual disease (MRD), as assessed by highly sensitive molecular and multiparameter flow cytometry assays in marrow samples at the time of clinical remission, may be a more significant and accurate predictor for leukemic persistence and recurrence than pathology alone. In fact, various recent studies have investigated the role of mutant* NPM1* as a tool for MRD assessment in AML [18–25]. The presence of MRD as determined by quantitation of* NPM1*-mutated transcripts after two cycles of chemotherapy in standard-risk AML was shown to be a powerful independent prognostic factor for disease relapse and overall survival in one study [22]. In another study, reduction in postinduction MRD based on peripheral blood* NPM1*-mutated transcripts had strong prognostic significance and predicted benefit from allogeneic stem cell transplant [18]. In the subset of patients with* FLT3* ITD, only age, white blood cell count, and 4-log reduction in peripheral blood MRD, but not* FLT3* ITD allelic ratio, were significantly associated with a higher cumulative incidence of relapse. Indeed, MRD may eventually be incorporated into routine practice as an important determinant of therapy response, modification, and need for transplant, similar to our current usage of AML cytogenetics and mutation information [13, 26].

Our patient's disease recurrence likely occurred from the AML founding clone or one of its subclones [27]. However, the lack of stored blood or marrow samples precludes confirmation of this aspect of clonal evolution. Nevertheless, the current definition of AML remission does not incorporate clonal evolution; rather, it relies heavily on morphology as discussed above. Notably, our patient experienced a significant spontaneous reduction in her overall leukemia disease burden lasting several weeks in the absence of any known therapy. While missed diagnosis due to patchy marrow involvement is a possibility, it should be noted that localized or patchy involvement is generally assumed to be very rare in AML unlike lymphoplasmacytic neoplasms or solid tumors [28]. A simultaneous improvement in hematologic parameters as well as* FLT3* ITD allelic ratio and then a worsening of these findings corresponding with frank relapse are highly indicative of true SR in this patient by current criteria. SR in AML patients are hypothesized to result from activation of innate host immune responses exerting direct antileukemic effects. Two factors resulting in immune activation, specifically infections and blood transfusion, have been repeatedly associated with the majority of SR cases. Unequivocal infections have been documented in 32 out of 45 (71.1%) SR patients, while 32 out of 39 (82.1%) SR patients had antecedent blood transfusion [4]. Although pneumonia (54.5%), bacteremia (24.2%), and skin/soft tissue infections (12.1%) are the most commonly cited infections [4], even atypical infections such as pulmonary aspergillosis, tuberculosis,* Pneumocystis jirovecii*, and infectious mononucleosis have been described [29–33]. Immune cell production of cytokines such as tumor necrosis factor-*α* (TNF-alpha) and interferon-*γ* (IFN-gamma) has been shown to directly inhibit myeloblast proliferation [34, 35]. Increased interleukin-2 (IL-2) levels also activate natural killer (NK) cell number and activity [34–36]. Hypergammaglobulinemia, representing humoral immune response, can occur secondary to antibody generation against blast antigens, antibody generation triggered by cytotoxic T-cell lymphocyte recognition of blasts, or antibody generation against infectious antigens with cross-reactivity to leukemic blasts [4, 36]. This may also contribute to increased antibody-mediated cytotoxicity via NK and cytotoxic T-cells and activate macrophages through better recognition, opsonization, or adhesion [37, 38]. In vitro experiments performed at 10-year follow-up of the longest sustained SR in AML containing the t (9; 11)(q22; q23) abnormality suggested that early immune effects were likely mediated by CD8 T-cell and humoral mechanisms while long-term remission was potentially mediated by NK-cells [38, 39].

Allogeneic blood product transfusion has also been linked to SR in AML. Because nonirradiated blood products contain potentially functional antileukemic lymphocytes, transfusion of such products can potentially induce antileukemic effects in a manner similar to graft-versus-leukemia [30, 40, 41]. The role of antileukemic antibodies or other factors in donor serum has also been speculated [42]. The current routine practice of using leukocyte depleted irradiated blood products in all patients with AML may have partly diminished the contribution of transfusions to SR and rendered SR an even rarer occurrence than in the past.

Cases of SR unrelated to transfusions or infections, as in our case, have also been noted previously [4, 43]. Potential other contributing factors including granulocyte-colony stimulating factor (GCSF) administration and hormonal changes (both of which could also impact on innate immune function) have been reported [43]. There is also data suggesting that certain biological subsets of AML may be more likely to undergo SR, specifically FAB subtypes M4/M5 which constitute about half of all reported cases of SR in AML, and certain cytogenetics [3, 4]. Of note, patients with* NPM1* mutant AML similar to our patient may also be predisposed towards SR [3, 44]. The presence of* NPM1* mutation, found in approximately 35% of AML cases, is known to confer favorable prognosis [45, 46]. When coexistent with* FLT3* ITD, the presence of* NPM1* mutation may partly mitigate the poor prognostic effects of* FLT3* ITD, particularly in patients with low* FLT3* ITD mutant disease levels, for unknown reasons [14, 47–50]. It is therefore of interest that, at the time of SR, our patient continued to have NPM1 mutant disease in the presence of very low levels of FLT3 ITD.

In conclusion, SR in AML is a rare but real and well-documented clinical phenomenon resulting in clinical complete remissions lasting for an average of 5–7 months and ranging from as short as 2 weeks to in excess of 100 months [4, 38, 51–54]. Identification of such patients along with systematic and comprehensive correlative studies, if feasible, will help elucidate the mechanistic underpinnings of this phenomenon in an otherwise fatal disease. The fact that SR occurs even in patients with high disease burden, relapsed/refractory disease, and AML with complex/adverse genetic abnormalities is a testament to the power of the immune system to inhibit leukemia. Prior attempts to activate the immune system via mechanisms previously documented in SR cases have so far had only limited success, for example, IL-2 for consolidation and relapsed/refractory AML therapy [4, 55, 56]. However, the advent of modern immunotherapeutic approaches beyond allogeneic stem cell transplant such as T-cell engaging antibody constructs and adoptive transfer of autologous chimeric antigen receptor (CAR) T-cells for AML therapy remain highly promising [57]. Hopefully, the mechanisms driving SR in AML can one day be unraveled and harnessed in combination with other therapeutic options. Although our patient was not able to receive experimental FLT3 tyrosine kinase inhibitor therapy for detectable FLT3 mutant disease in the setting of morphologic CR, the future use of FLT3 inhibitors for the treatment of minimal molecular residual disease and as a complementary approach to immunotherapy is also appealing.

## Figures and Tables

**Table 1:** Laboratory and molecular results over the course of patient's disease. AML: acute myeloid leukemia, AR: allelic ratio, BM: bone marrow, CR: complete remission, FLT3 ITD: FMS-like tyrosine kinase 3 internal tandem duplications, Hgb: hemoglobin, NPM1: nucleophosmin-1, PB: peripheral blood, SR: spontaneous remission, and WBC: white blood cell.

	Diagnosis Day 1	CR Day 38	Relapse Day 270 (BM-1)	First SR Day 277 (BM-2)	Second SR Day 284 (BM-3)	Second relapse Day 312
WBC (× 10^9^/L)	240	8.7	3.3	3.51	4.72	29.9
Hgb (g/dL)	6.2	8.2	11.4	12.2	12.4	11.8
Platelets (× 10^9^/L)	119	483	115	150	171	63
PB blasts (%)	74%	None	None	None	None	16%
BM blasts (%)	73%	None	64%	3%	2%	84%
Flow cytometry	Abnormal	No AML	Abnormal	No AML	No AML	Abnormal
BM Cytogenetics	Normal	N.A.	del (15q) in 19/20 cells	del (15q) in 1/20 cells	del (15q) in 6/20 cells	del (15q) in 20/20 cells and del (16q) in 1/20 cells
*NPM1* mutation	+ BM	+ BM	+ BM	+ PB/+ BM	+ BM	+ BM
*FLT3* ITD mutation	+ BM (AR N.A.)	− BM	+ BM AR 0.36	− BM/+ PB AR 0.005	+ BM AR 0.01	+BM AR 1.99
